# Association analysis between hyperuricemia and long term mortality after acute coronary syndrome in three subgroups of patients

**DOI:** 10.1016/j.dib.2018.01.101

**Published:** 2018-02-05

**Authors:** Adriana Lopez-Pineda, Alberto Cordero, Concepción Carratala-Munuera, Domingo Orozco-Beltran, Jose A. Quesada, Vicente Bertomeu-Gonzalez, Vicente F. Gil-Guillen, Vicente Bertomeu-Martinez

**Affiliations:** a Cardiology Department, Hospital of San Juan de Alicante, San Juan De Alicante, Spain; b Catedra de Medicina de Familia, Clinical Medicine Department, Miguel Hernandez University, San Juan de Alicante, Spain; c Centro de Investigación Biomédica en Red de Enfermedades Cardiovasculares (CIBERCV), Hospital Clínico Universitario de Santiago de Compostela-SERGAS. Instituto de Investigación Sanitaria de Santiago de Compostela (IDIS), Santiago de Compostela, Spain; d Clinical Medicine Department, Miguel Hernandez University, San Juan de Alicante, Spain; e Centro de Investigación Biomédica en Red de Enfermedades Cardiovasculares (CIBERCV), Fundación para la Investigacion del Hospital Clínico de la Comunidad Valenciana (Fundación INCLIVA), Valencia, Spain

## Abstract

These data are linked to the research article, entitled Hyperuricemia as a prognostic factor after acute coronary syndrome published in Atherosclerosis. Data from patients admitted for acute coronary syndrome between 2008 and 2013 were collected during the hospitalization, and a follow-up until endpoint or end of study was carried out. Multivariate analysis of variables associated with long term mortality after acute coronary syndrome in patients stratified by the presence of diabetes, hypertension or kidney failure is provided in this article.

**Specifications Table** *[please fill in right-hand column of the table below]*

**Table:** 

Subject area	*Medicine*
More specific subject area	*Cardiology*
Type of data	*Table*
How data was acquired	*Clinical variables were obtained from patient medical report.*
	*Multivariate analysis was performed with SPSS 22.0*
Data format	*Analyzed*
Experimental factors	*Clinical variables and serum uric acid level were measured during hospitalization after acute coronary syndrome*
	*Patients were followed-up until event or end of study*
Experimental features	*Multivariate analysis was adjusted using the likelihood ratio test for variables selection procedure*
Data source location	*San Juan de Alicante University Hospital, San Juan de Alicante, Spain*
Data accessibility	*Within this article*

**Value of the data**

• These data help to clarify if a high serum uric acid level is independently associated with long-term mortality after acute coronary syndrome or simply indicates the presence of three important cardiovascular risk factors.
• As kidney failure is known as a cardiovascular risk factor and might influence the serum uric acid level [1], the independent association between hyperuricemia and total and cardiovascular mortality after acute coronary syndrome was analyzed in patients without kidney disease.
• As diabetes is a cardiovascular risk factor and its influence in serum uric acid level is being investigated [2], [3], the independent association between hyperuricemia and total and cardiovascular mortality after acute coronary syndrome was analyzed in patients without diabetes.
• As hypertension is an important cardiovascular risk factor and evidence shows that hyperuricemia and hypertension are independently associated [4], the independent association between hyperuricemia and total and cardiovascular mortality after acute coronary syndrome was analyzed in patients without hypertension.

## 1 Data

Data about the association of hyperuricemia and other factors with total and cardiovascular mortality after acute coronary syndrome in patients without kidney failure are presented in Table 1. The same information in non-diabetic and non-hypetensive patients are showed in Table 2 and Table 3, respectively.

**Table 1:** Multivariate analysis of variables associated with long term mortality in the subgroup of patients without kidney disease (independent predictors of outcome). Adjusted for age, sex, cardiovascular risk factors (body mass index, hypertension, smoking habit, diabetes and dyslipidemia), glomerular filtration rate, previous coronary heart disease, heart failure or stroke, as well as medical treatments at discharge (clopidogrel, prasugrel, ticagrelor, dual antiplatelet treatment, betablockers, ACEI/ARB, statins, diuretics, espironolactone/eplerenone, nitrates, oral antidiabetics).

	**HR (95%CI); *p*-value**	
**Independent Variables**	**Cardiovascular mortality**	**All-cause mortality**

**Age > 75**	2.96 (1.73–5.07); *p* < 0.001	3.00 (1.94–4.63); *p* < 0.001
**Charlson > 4**	3.00 (1.80–5.00); *p* < 0.001	2.73 (1.79–4.15); *p* < 0.001
**Hyperuricemia**	1.94 (1.16–3.24); *p* = 0.011	1.62 (1.06–2.47); *p* = 0.025
**Non-revascularization**	5.59 (3.34–9.35); *p* < 0.001	4.12 (2.67–6.35); *p* < 0.001
**Previous HF**	ns	5.35 (1.90–15.11); *p* = 0.002
**GRACE score**	1.01 (1.00–1.02); *p* = 0.003	1.01 (1.00–1.02); *p* < 0.001

Goodness-of-fit indicators: *n* = 851, LRT = 95.9, *p* < 0.001.

HR: hazard ratio; CI: confidence interval; ns: non-significant; HF: heart failure; GRACE: Global registry of acute coronary events.

**Table 2:** Multivariate analysis of variables associated with long term mortality in the subgroup of patients without diabetes (independent predictors of outcome). Adjusted for age, sex, cardiovascular risk factors (body mass index, hypertension, smoking habit, diabetes and dyslipidemia), glomerular filtration rate, previous coronary heart disease, heart failure or stroke, as well as medical treatments at discharge (clopidogrel, prasugrel, ticagrelor, dual antiplatelet treatment, betablockers, ACEI/ARB, statins, diuretics, espironolactone/eplerenone, nitrates, oral antidiabetics).

	**HR (95%CI); *p*-value**	
**Independent Variables**	**Cardiovascular mortality**	**All-cause mortality**
**Age > 75**	3.32 (1.77–6.20); *p* < 0.001	3.70 (2.35–5.81); *p* < 0.001
**Charlson > 4**	2.29 (1.12–4.66); *p* = 0.023	2.83 (1.64–4.89); *p* < 0.001
**Hyperuricemia**	ns	ns
**Previous HF**	2.81 (1.20–6.56); *p* = 0.017	3.35 (1.61–6.95); *p* = 0.001
**Non-revascularization**	3.03 (1.77–5.18); *p* < 0.001	2.35 (1.48–3.74); *p* < 0.001
**GRACE score**	1.01 (1.00–1.02); *p* = 0.003	ns
**IHD**	2.70 (1.57–4.65); *p* < 0.001	1.71 (1.11–2.62); *p* = 0.014
**Female**	1.82 (1.01–3.25); *p* < 0.001	ns
**No statins at discharge**	ns	2.74 (1.50–5.02); *p* = 0.001

Goodness-of-fit indicators: *n* = 716, LRT = 105.5, *p* < 0.001.

HR: hazard ratio; CI: confidence interval; ns: non-significant; HF: heart failure; IHD: ischemic heart disease; GRACE: Global registry of acute coronary events.

**Table 3:** Multivariate analysis of variables associated with long term mortality in the subgroup of patients without hypertension (independent predictors of outcome). Adjusted for age, sex, cardiovascular risk factors (body mass index, hypertension, smoking habit, diabetes and dyslipidemia), glomerular filtration rate, previous coronary heart disease, heart failure or stroke, as well as medical treatments at discharge (clopidogrel, prasugrel, ticagrelor, dual antiplatelet treatment, betablockers, ACEI/ARB, statins, diuretics, espironolactone/eplerenone, nitrates, oral antidiabetics).

	**HR (95%CI); p-value**	
**Independent Variables**	**Cardiovascular mortality**	**All-cause mortality**
**Non-revascularization**	5.42 (1.57–18.67); *p* = 0.007	3.21 (1.17–8.82); *p* = 0.024
**Hyperuricemia**	3.58 (1.18–10.81); *p* = 0.023	ns
**Age > 75**	14.55 (4.61–45.94); *p* < 0.001	4.39 (1.94–9.94); *p* < 0.001
**GRACE score**	ns	1.01 (1.00–1.02); *p* = 0.004

Goodness-of-fit indicators: *n* = 362, LRT = 33.6, *p* < 0.001.

HR: hazard ratio; CI: confidence interval; ns: non-significant; GRACE: Global registry of acute coronary events.

## 2 Experimental design, materials and methods

Lopez-Pineda et al. [5] found that a serum uric acid level above the normal range was independently associated with both total and cardiovascular mortality as well as major cardiovascular events in medium/long-term after acute coronary syndrome. Additional multivariate analysis with patients without the presence of risk factors such as diabetes, hypertension or kidney failure was performed in order to better asses the influence of these risk factors.

All consecutive patients admitted for an acute coronary syndrome between December 2008 and December 2013 were included. We collected demographic characteristics, cardiovascular risk factors, previous medical history, laboratory data during the hospitalization, vital signs on admission, treatment, and diagnosis at discharge from all patients. Serum uric acid level was routinely measured following overnight fasting from peripheral venous blood samples within the first 24–48 h of hospitalization. Colorimetry and uricase method were used to measure it. According to the local laboratory reference range, hyperuricemia was defined as SUA higher than 7 mg/dL (420 μmol/L) in men and 5.7 mg/dL (342 μmol/L) in women. The glomerular filtration rate (GFR) was estimated on admission from serum creatinine values with the Modification of Diet in Renal Disease (MDRD) study equation (31). GFR values less than 60 mL/min/m^2^ were considered to indicate kidney failure. We defined comorbid hypertension and diabetes mellitus according to previous diagnosis on patient medical reports or if the patient was receiving specific therapies. Participants with HbA1c greater than or equal to 6.5% and no previous diagnosis were codified as diabetics. After discharge, participant follow-up was carried out in order to obtain clinical status and outcome events from study inclusion to October 2016 or first observed outcome event. The primary endpoint was cardiovascular mortality. All-cause mortality was one of the secondary endpoints. Further details of methodology have been previously published [5].

### 2.1 Statistical analysis

Data were processed with SPSS 22.0 and STATA 14.0 software. Multivariate analysis was adjusted using the likelihood ratio test for variables selection procedure. A selective stepwise-all variables with a P value < 0.05 were assessed in a step-backward model. The results are presented as hazard ratios (HR) with 95% confidence intervals (CI). The threshold for establishing statistical significance was p < 0.05.

### 2.2 Patients without kidney disease

859 patients of total sample (76.8%) had GFR ≥ 60 ml/min/1.72 m^2^ and the multivariate analysis, adjusted for age, sex, cardiovascular risk factors (body mass index, hypertension, smoking habit, diabetes and dyslipidemia), glomerular filtration rate, previous coronary heart disease, heart failure or stroke, as well as medical treatments at discharge (clopidogrel, prasugrel, ticagrelor, dual antiplatelet treatment, betablockers, ACEI/ARB, statins, diuretics, espironolactone/eplerenone, nitrates, oral antidiabetics), showed the association between the hyperuricemia factor and cardiovascular and all-cause mortality in this subgroup of patients (Table 1).

### 2.3 Patients without diabetes

729 patients of total cohort (65.1%) were non-diabetic patients the multivariate analysis, adjusted for age, sex, cardiovascular risk factors (body mass index, hypertension, smoking habit, diabetes and dyslipidemia), glomerular filtration rate, previous coronary heart disease, heart failure or stroke, as well as medical treatments at discharge (clopidogrel, prasugrel, ticagrelor, dual antiplatelet treatment, betablockers, ACEI/ARB, statins, diuretics, espironolactone/eplerenone, nitrates, oral antidiabetics), showed the association between the hyperuricemia factors and cardiovascular and all-cause mortality in this subgroup of patients (Table 2).

### 2.4 Patients without hypertension

368 (32.9%) patients of the total sample were non-hypertensive patients and the multivariate analysis, adjusted for age, sex, cardiovascular risk factors (body mass index, hypertension, smoking habit, diabetes and dyslipidemia), glomerular filtration rate, previous coronary heart disease, heart failure or stroke, as well as medical treatments at discharge (clopidogrel, prasugrel, ticagrelor, dual antiplatelet treatment, betablockers, ACEI/ARB, statins, diuretics, espironolactone/eplerenone, nitrates, oral antidiabetics), showed the association between the hyperuricemia factor and cardiovascular and all-cause mortality (Table 3).
