# Supplementary material for: Association analysis between hyperuricemia and long term mortality after acute coronary syndrome in three subgroups of patients
Source: Data Brief. 2018 Feb 5;17:885–9. doi: 10.1016/j.dib.2018.01.101 (PMC5834648; doi:10.1016/j.dib.2018.01.101)
Supplement: Supplementary file 1 — Supplementary material [file mmc1.docx]

Conflict of Interest

No conflict of interest.
